# Supplementary material for: Biomimetic light-harvesting funnels for re-directioning of diffuse light
Source: Nat Commun. 2018 Feb 14;9:666. doi: 10.1038/s41467-018-03103-4 (PMC5812990; doi:10.1038/s41467-018-03103-4)
Supplement: Supplementary file 1 — Supplementary Information [file 41467_2018_3103_MOESM1_ESM.pdf]

Supplementary Information

Biomimetic light-harvesting funnels for re-directioning of diffuse light

Pieper et al.

Supplementary Figures

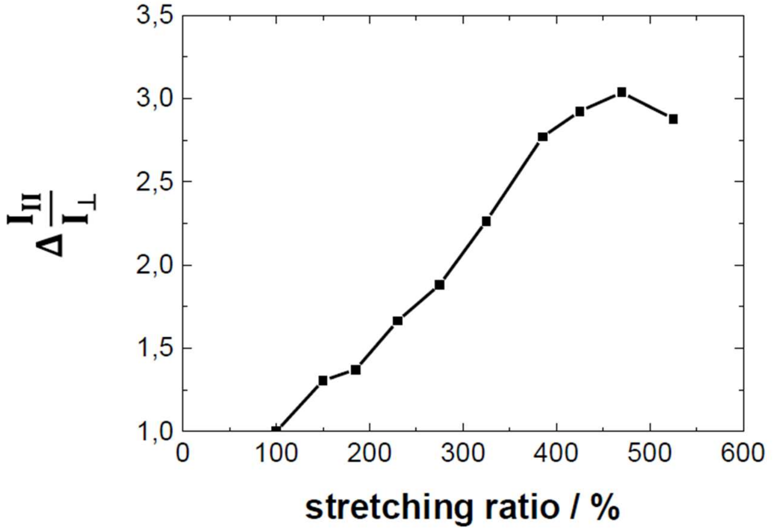

Supplementary Figure 1 | Dye reorientation of Cumarin 6 in PVA as a function of polymer expansion.

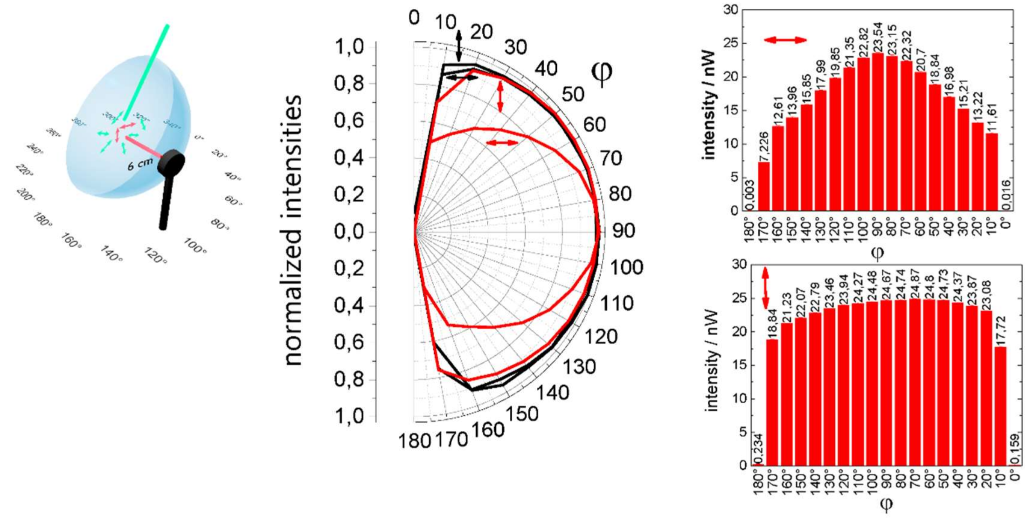

Supplementary Figure 2 | Experimental raw intensities observed after donor excitation and with aligned acceptors (red) and random acceptors (black), respectively. Arrows indicate foil orientation.

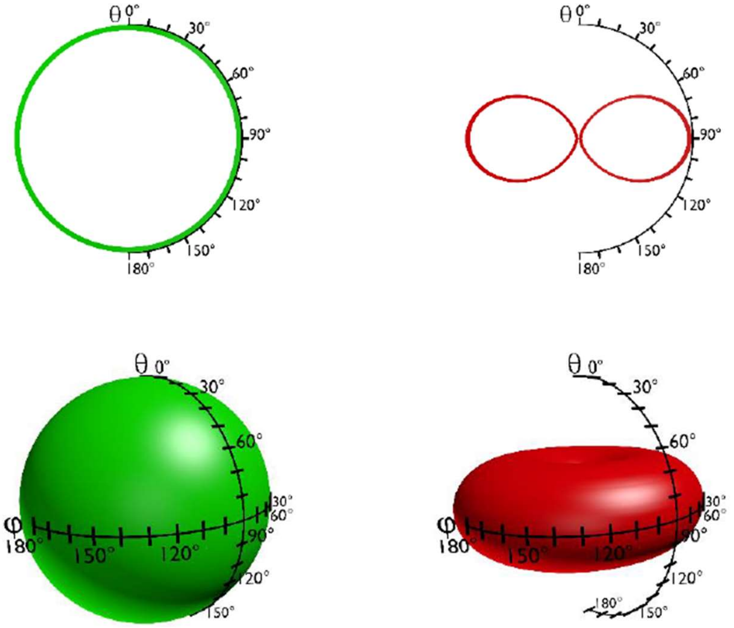

Supplementary Figure 3 | Illustration of weighing factors for transforming two-dimensional into three-dimensional angle distributions.

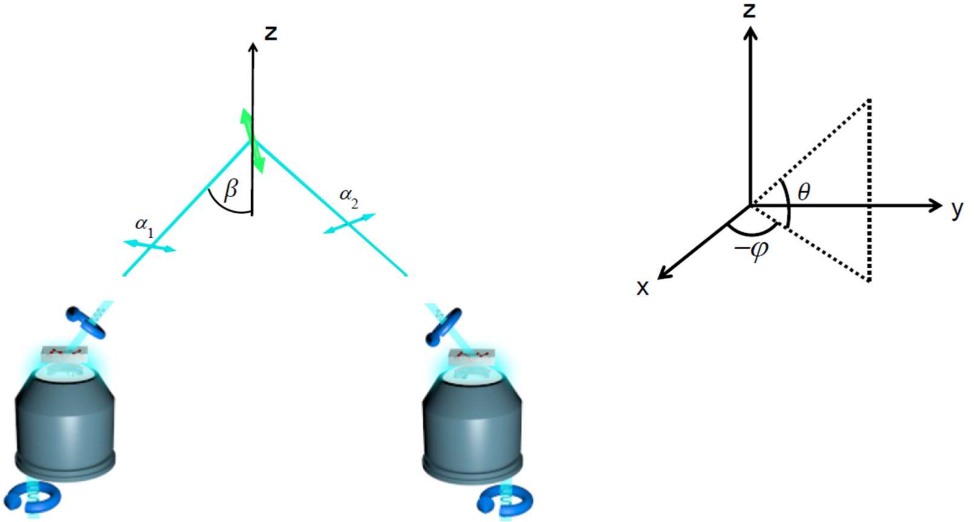

Supplementary Figure 4 | Schematic representation of the angles in the reconstruction of three dimensional molecule orientations.

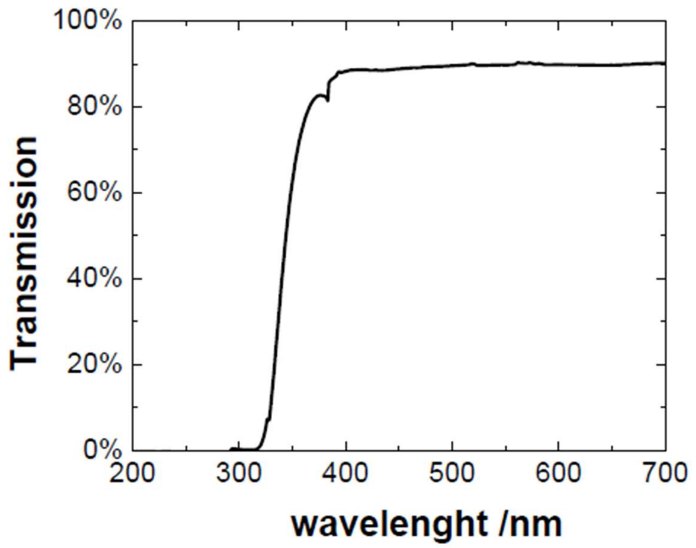

Supplementary Figure 5 | Transmission spectrum of Glass Hemisphere.

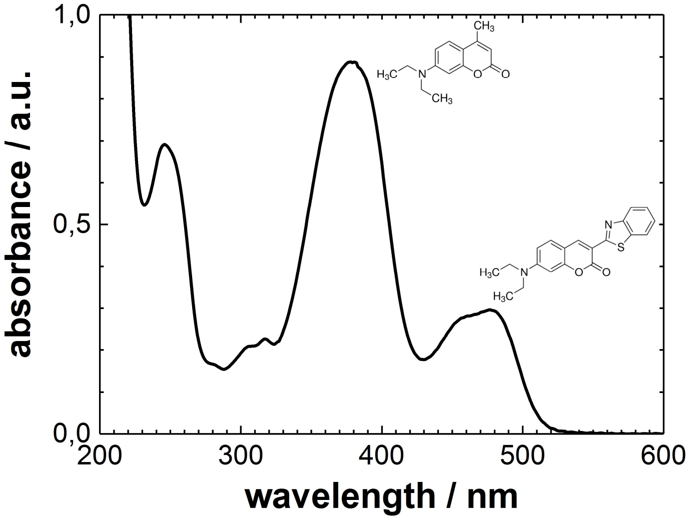

Supplementary Figure 6 | Absorption spectrum of a single foil with aligned acceptors for light incident at 90°. Thickness ~ 30 µm.

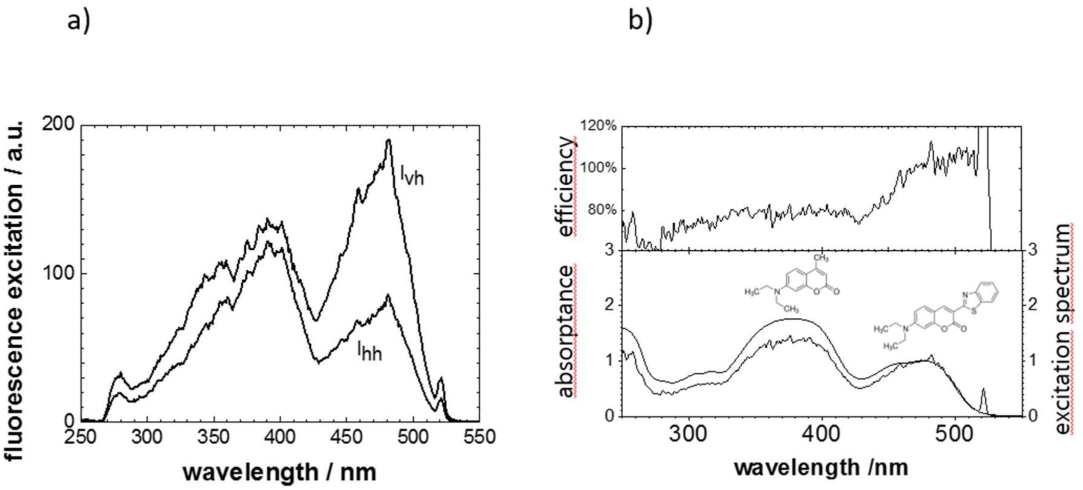

Supplementary Figure 7 | Absorptance and Excitation spectra of polymer foils with aligned acceptors. Please note that the absorptance of a sample is not identical to the absorbance. For details see Gutierrez et al. (2016)[1].

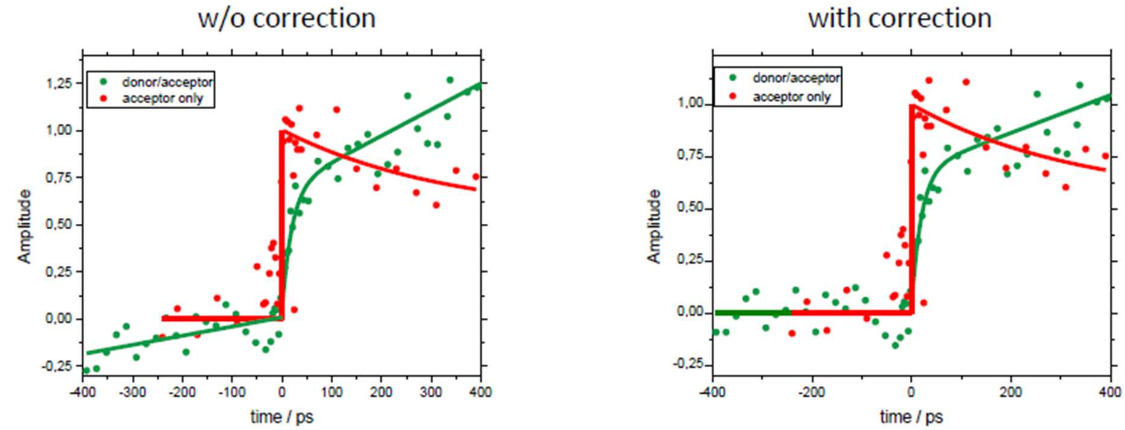

Supplementary Figure 8 | Linear background correction of long component in donor/acceptor Pump-Probe Data.

Supplementary Tables

Supplementary Table 1 | Reorientation capability of various dyes in expanded PVA

Dye	$\Delta\ I_{\parallel}/I_{\perp}$	% Expansion	$\lambda^{max}_{Absorption}$ /nm	$\lambda^{max}_{Emission}$ /nm	Φ_{Fl}
Coumarin 6	3.39	400-500	474	504	0.78[2] (in EtOH, higher in polymers)
Coumarin 6	2.71	200	474	504	0.78[2] (in EtOH, higher in polymers)
Cresyl Violet Perchlorate (~10^{-5}M)	3.15	250	606	626	0.51[3] (in EtOH)
Cresyl Violet Perchlorate (~10^{-4}M)	1.92	250	606	632	0.51[3] (in EtOH)
Acridine yellow	4.49	270	471	502	0.47[3] (in EtOH)
Coumarin 1	1.28	400	377	432	0.50[2]/0.73[4] (in EtOH)
Coumarin 1	1.17	~200	377	432	0.50[2]/0.73[4] (in EtOH)
Atto 390 (various concentrations)	0.91-1.07	220-250	388	455	0.90[5] (in H_2O)
Atto 425 (various concentrations)	0.98-1.05	200-230	436	478	0.90[5] (in H_2O)
Atto 465 (various concentrations)	1.16-1.47	220-270	467	490	0.55[5] (in H_2O)
Rhodamin 6G	1.14	220	533	555	0.95[6] (in EtOH)

Supplementary Table 2 | Measured, extrapolated and theoretical ideal two- and three-dimensional photon angle distributions of aligned acceptor emission

Detector angle φ	Acceptor emission observed after donor excitation (l_{Exc} = 375 nm, Φ = 20°) / nW	Weighing Factor for 3D intensity distribution	Weighed by 3D-factor / nW	Normalized acceptor emission observed after direct excitation (l_{Exc} = 485 nm, Φ = 90°)	Weighed by 3D-factor	Theoretical emission of ideally aligned acceptors	Weighed by 3D-factor
0	0,003 / 9,91[1]	0,00	0 / 0[1]	0,23	0,00	0,00	0,00
10	7,226 / 11,26[1]	0,29	2,09 / 3,25[1]	0,33	0,10	0,03	0,01
20	12,61	0,57	7,17	0,43	0,24	0,12	0,07
30	13,96	0,83	11,60	0,53	0,44	0,25	0,21
40	15,85	1,07	16,94	0,63	0,67	0,41	0,44
50	17,99	1,27	22,91	0,77	0,98	0,59	0,75
60	19,85	1,44	28,58	0,81	1,17	0,75	1,08
70	21,35	1,56	33,35	0,90[2]	1,41	0,88	1,38
80	22,82	1,64	37,36	0,96[2]	1,57	0,97	1,59
90	23,54	1,66	39,13	0,99[2]	1,65	1,00	1,66
100	23,15	1,64	37,90	0,99[2]	1,62	0,97	1,59
110	22,32	1,56	34,86	0,95[2]	1,48	0,88	1,38
120	20,7	1,44	29,80	0,89	1,28	0,75	1,08
130	18,84	1,27	23,99	0,8	1,02	0,59	0,75
140	16,98	1,07	18,14	0,73	0,78	0,41	0,44
150	15,21	0,83	12,64	0,59	0,49	0,25	0,21
160	13,22	0,57	7,52	0,43	0,24	0,12	0,07
170	11,61 / 11,23[1]	0,29	3,35 / 3,24[1]	0,27	0,08	0,03	0,01
180	0,016 / 9,24[1]	0,00	0 / 0[1]	0,1	0,00	0,00	0,00
Average	15.21	1	19.33				
Fraction in 90°±50°			88%		90%		96%
Fraction in 90°±60°			94%		96%		99%

[1] Value observed from linear extrapolation

[2] Values estimated by scaling values observed with Φ = 20° excitation

Supplementary Notes

Supplementary Note 1

Screening of various dyes for molecular re-orientation in expanding polyvinyl alcohol (PVA)

Re-orientation of dye orientation in expanding PVA was tested with Coumarine 6, Acridine Yellow, Cresyl Violet Perchlorate, Rhodamine 6G, ATTO 390, ATTO 425, ATTO 465 and Coumarine 1. Dye orientation in the host matrix was screened using a Cary Eclipse Fluorescence spectrometer from VARIAN. Fluorescence excitation spectra were taken from unstreched and stretched dye containing foils prepared as described in the material and methods section and by using vertically (v) or horizontally (h) polarized excitation and detection (Manual Polarizer Accessory from VARIAN). Thus, four intensities I_{hh}, I_{vh}, I_{vv} and I_{hv} were detected for each possible combination. The first index represents the polarization of the excitation while the second represents the polarization of the detection, respectively. For example, I_{vh}, was the intensity observed with excitation polarization perpendicular and detection polarization parallel to the polymer expansion direction, which was always horizontal. Intensities observed with unpolarized excitation can be calculated by averaging the intensities observed with parallel and perpendicular excitation. Thus, the light intensity with parallel detection but unpolarized excitation is $I_{\parallel} = (I_{hh}+I_{vh})/2$ and the corresponding value with perpendicular detection is $I_{\perp} = (I_{hv}+I_{vv})/2$, respectively. When reorientation occurs, the intensity measured with detection polarization parallel to the expansion direction, $I_{\parallel}$, becomes larger than the corresponding intensity detected perpendicular to the expansion direction, $I_{\perp}$. In Supplementary Table 1 relative changes in the ratio of these two intensities, $\Delta\ I_{\parallel}/I_{\perp}$, were plotted for each dye along with the actually percentage of polymer expansion. The ratio change $\Delta\ I_{\parallel}/I_{\perp}$ was calculated from the ratio $I_{\parallel}/I_{\perp}$ observed with expanded polymer divided by the corresponding ratio, $I_{\parallel}/I_{\perp}$, observed with non-expanded polymer. In addition, the table gives the wavelengths for the absorption and emission maxima, $\lambda^{max}_{Absorption}$ and $\lambda^{max}_{Emission}$,and some literature fluorescence quantum yields, Φ_{Fl}.

The data in Supplementary Table 1 demonstrate that during polymer expansion significant molecular re-orientation was observed for Coumarine 6, Acridine Yellow and Cresyl Violet Perchlorate. In contrast, Rhodamine 6G, ATTO 390, ATTO 425, ATTO 465 and Coumarine 1 displayed significantly less molecular re-orientation. Supplementary Figure 1 shows the degree of re-orientation of Coumarin 6 as a function of polymer expansion. These data demonstrate that after about 300% expansion no significant further re-orientation could be observed for Coumarin 6 in PVA.

We decided to use the pair Coumarin 1 as donor and Coumarin 6 as acceptor for the proof-of principle since Coumarin 6 has a high emission quantum yield, is significantly aligned during expansion, has a perfect spectral overlap for energy transfer with Coumarin 1 and because Coumarin 1 basically keeps a random orientation during expansion.

In a next step we screened a multitude of donor-acceptor concentrations and ratios in PVA. For each concentration and ratio value we determined the absorptivity at the donor excitation wavelength, the absolute intensity emitted by the acceptors and the change in $I_{\parallel}/I_{\perp}$ during polymer expansion and observed after donor excitation. We decided to use for in Figure 2 and 3 in the main

manuscript the concentrations corresponding to ~17 mM Coumarin 1 and ~1.9 mM aligned Coumarin 6 in the stretched foils. Using these concentrations we observed well aligned acceptors, high energy transfer efficiencies, high absorption at the donor excitation wavelength and little reabsorption at acceptor emission wavelengths.

Supplementary Note 2

Angle dependent direct power measurements (Photo goniometer)

As described in the methods section, the foils were directly attached to a glass hemisphere for the direct power measurements (Figure 2 a in the main text). This minimizes the influence of refraction effects that would otherwise occur at the foil air surface. Foils with aligned acceptors were either attached with dipole orientations parallel (∥, horizontal) or perpendicular (⊥, vertical) to the detector angle range of φ = 0-180° (cf. Figure 2 a). Donor excitation (375 nm) was at angles nearly perpendicular to the horizontal detector angle range (Θ = 20°, green line in Figure 2 a, angles defined in the same way as in Supplementary Figure 3). Direct acceptor excitation (485 nm) was done perpendicularly to the foil surface (Θ=90°, parallel to the red line in Figure 2 a), as this allowed to better determine the emission anisotropy due to photoselection (preferential excitation of molecules that re-emit in directions parallel to the excitation, Figure 1 c and 2 d).

Supplementary Figure 2 shows the absolute detected light intensities from the acceptors with nearly perpendicular donor excitation (Θ=20°, green line in Figure 2 a). This data was observed with unstretched and streched foils containing ~17 mM Coumarin 1 and ~1.9 mM aligned Coumarin 6 perpendicular (⊥,↕) or parallel (∥,↔) to the detector angle range φ = 0-180° (cf. Figure 2 a). When using beam powers in the range of ~10 to 140 µW (corresponding to intensities of about abut 1-14 mW·cm^{-2}) we observed for most samples a linear dependence of harvested and re-emitted light intensity. The data were obtained using a calibrated powermeter with 0,49 cm^2 detection area at a distance of 6.1 cm from the light harvester and a total irradiation intensity of I_{Exc} = 94 µW using a collimated beam of 375 nm in wavelength. Thus the measured intensities correspond to the angle dependent fraction measured in a fraction of F_{Area} = 0,49 cm^2 / 468 cm^2 = 0.105% of the entire sphere surface of 6.1 cm radius around the irradiation point.

Re-direction quantum efficiency

The data in Supplementary Figure 2 allows determining the fraction of photons emitted from aligned acceptors within an angle range suitable for total internal reflection wave guiding. Typically, the refractive index of transparent polymers such as PMMA or glasses is on the order of 1.5 but materials with indices of 1.9 or even higher exist as well. For such materials total reflection occurs at angles smaller than 90°-arcsin(1/n) = 48° or 58° at the surface with air, respectively. Supplementary Figure 2 and Supplementary Table 2 directly shows the measured powers at various angles with respect to the aligned acceptors (Figure 2 a). The angle range for total reflection corresponds to the data detected at angles of ~40°-140° and 30°-150° for refractive indices of 1.5 and 1.9, respectively (see also Figure 2 b-f). Supplementary Table 2 also contains linearly

extrapolated values for angles close to 0° and 180° that could not be accurately determined experimentally due to residual refraction observed for very small angles with the foil hemi sphere interface. Note that linear interpolation is conservative for determining escape cone losses as the expected distribution should decrease more quickly at small angles when assuming a $\cos^2$ intensity distribution. In addition, the table also contains angle dependent powers observed with direct excitation of aligned acceptors and an ideal $\cos^2$ distribution expected for perfectly aligned acceptors. These three distributions are used for Figure 2f in the main paper (i,ii and iii). All directly measured values are indicated by a direct line in Figure 2 b-f while extrapolated or calculated values are indicated by a dotted line.

For determining the photons totally reflected in three-dimensions it has to be considered, that polar coordinates of Θ = 90° corresponds to a much larger three-dimensional angle range in φ than Θ = 0° or Θ = 180° (Supplementary Figure 3). While Θ = 90° corresponds to a full "equatorial" φ angle range in a three-dimensional distribution, Θ =0° and Θ = 180° correspond only to a single point direction ("North" and "south" pole, Supplementary Figure 3). To account for the fact that in the entire three dimensional emission profile the values measured at different angles Θ correspond to different contributions to the total emission intensity, the observed values have to be weighed by the normalized factor $\sin \Theta / \int \sin \Theta d\Theta$. In Supplementary Table 2 these angle dependent weighing factors (column 3) are summarized along with the directly measured powers (column 2) and the powers weighed by these factors (column 4).

The weighed photon fractions of the three distributions i,ii and iii demonstrate that 88%, 90% and 96% fall into an angle range suitable for total reflection wave guiding in materials with a refractive index of 1.5 and 94%, 96% and 99% fall into an angle range for materials with a refractive index of 1.9, respectively (Supplementary Table 2). The range suitable for total reflection wave guiding in materials with a refractive index of 1.5 is indicated by the red areas in the two-dimensional representation in Figure 2 f.

Supplementary Note 3

Estimate of energy transfer efficiency by calculations of the Förster Energy transfer rate and Förster Radius

A common way to estimate energy transfer efficiencies is to calculate the energy transfer rates, k_{ET}, and Förster distances, R_0, directly from the donor and acceptor spectra as well as distances. The Förster distance is the distance for a given pair of donor acceptor molecules at which 50% efficient energy transfer occurs. It can be calculated from easily accessible experimental data using the following equation[9]

$$R_0 = \sqrt[6]{\frac{9\ln(10)\kappa^2\phi_{Fl}^D}{128\pi^5 N_A n^4}\int_0^\infty I_{Fl,norm}^D(\lambda)\varepsilon_A(\lambda)\lambda^4 d\lambda} \qquad (1)$$

Here, the factor κ^2 depends on the relative donor-acceptor orientation, Φ_{Fl}^{D} is the fluorescence quantum yield of the donor molecules, n is the refractive index of the dye host material PVA and N_A is the Avogadro constant . The overlap integral, $J = \int_0^\infty I_{Fl,norm}^{D}(\lambda)\varepsilon_A(\lambda)\lambda^4 d\lambda$ is calculated from the normalized fluorescence spectrum of the donor $I_{Fl,norm}^{D}(\lambda)$ and the absorption spectrum of the acceptor $\varepsilon^A(\lambda)$. (Figure 3 a). In the case of Coumarin 1 and Coumarin 6 the resulting value is

$J = 1{,}75 \cdot 10^{-22}\, m^6 mol^{-1}$. Using a value of $\Phi_{Fl}^{D} = 0.73$ (Supplementary Table 1) and assuming randomly oriented molecules (κ^2 = 2/3) eq. 2 yields a Förster distance of $R_0 = 4.94\, nm$.

When the actual donor-acceptor distance, r, is known the quantum efficiency of energy transfer, Φ_{ET}, can be calculated using

$$\Phi_{ET} = \frac{1}{1 + (\frac{r}{R_0})^6} \qquad (2)$$

Assuming equal distances between the donor molecules a concentration of C = 17 mM correspond to an interval between the donors of $r \sim 1/\sqrt[3]{C} = 4.6$ nm. Thus, the center to center distance of additionally added acceptor molecule to its nearest donor is, regardless of the acceptor concentration, on the order of half that value, i.e. ~2.3 nm. Considering the dimensions of Coumarin 1 and Coumarin 6 this corresponds already to an inter-pigment distance on the order of only about 1.5 nm. Thus also other energy transfer mechanisms might not be negligible that occur for overlapping electronic wave function. A more precise determination would also need to take into account that for non-equally distributed molecules the average distance of acceptors to their *nearest* donors is smaller than average donor-acceptor distance. In any case, the distances are significantly smaller than the Förster Radius and can be optimized by varying donor-acceptor concentrations and ratios. A nearest donor-acceptor distance of ~2.3 nm, for example, corresponds to an energy transfer quantum yield of 99% (eq. 3).

The rate of the energy transfer can be calculated from the following equation:

$$k_{ET} = 0.529 \cdot \frac{\Phi_{Fl}^{D}}{\tau_{S_1}^{D}} \cdot \frac{1}{r^6}\kappa^2 \cdot \frac{1}{n^4 N_A} \cdot \int I_{Fl,norm}^{D}(\lambda)\, \varepsilon^A(\lambda)\, \lambda^4\, d\lambda \qquad (3)$$

Using for the donor lifetime the value $\tau_{S_1}^{D} = 3\text{ ns}$, for $n = 1.51$ and again an orientation factor for random orientations, $\kappa^2 = \frac{2}{3}$, eq. 5 yields for a nearest donor-acceptor distance of 2.3 nm a rate of $k_{ET} = 3.23 \cdot 10^{10}\ s^{-1}$. This corresponds to a time constant of 31 ps, which is in very good agreement with the fastest, experimentally observed value (see below). This further confirms efficient energy transfer as observed in the absolute power measurements.

Supplementary Note 4

Combined energy transfer and fluorescence quantum efficiency and reabsorption determined from angle dependent absolute power measurements and absorption spectroscopy

When determining the absolute irradiation and re-emitting powers directly at the samples further factors such as absorption by the glass-hemisphere, the absolute absorption by the sample itself and the transmission of the detection filter have to be considered. The optical density for the full optical path length through the hemi sphere showed that it transmits T_{375nm} = 83% of the excitation light at 375 nm (Supplementary Figure 5). This value already contains about 4% reflection losses at the air / glass transition as the glass hemi sphere was not coated with an anti-reflex coating.

In addition, Supplementary Figure 6 shows the absorption measurement of a single polymer foil with aligned acceptors and measured at 90° incident angle. However, for an incident angle of 20°, as used for detecting the emission anisotropy measurements in Figure 2, the optical path length through the foil is correspondingly longer. For light hitting a single foil at angles, Θ, smaller than 90° the optical path length through the foil increases by a factor of 1/sin Θ. Thus, also the optical density increases by the same factor. For an angle of 20° this corresponds to a factor in the optical path length of 1/sin 20° = 2.92. The optical density of a single foil at 375 nm and Θ=90° is 0.88 (Supplementary Figure 6). Thus, the optical density at 375 nm and Θ=20° is OD = 0.88 · 2.92 = 2.58. The transmitted intensity, I, relates to the initial intensity, I_{Exc}, by $I = I_{Exc} / 10^{OD} = I_{Exc} \cdot 0.0026$. Thus, Φ_{Abs} = 99,74% of the irradiated light was absorbed by the stretched foil. Please note that also in an arrangement as shown in Figure 4a light is typically hitting the foils at angles significantly smaller than 90°. For example, here light hits only a single foil if it is incident at angles below 15°. Light of larger angles hits at least two foils. The detection filter transmits about $T_{Detection} = \frac{\int_{400\,nm}^{700\,nn} I_{Fl}(\lambda)\cdot T_{Filter}(\lambda)d\lambda}{\int_{400\,nm}^{700\,nn} I_{Fl}(\lambda)d\lambda} = 43.6\%$ in the spectral emission range of the acceptor (Figure 3a). Here, $I_{Fl}(\lambda)$, is the emission spectrum of the acceptor and, $T_{Filter}(\lambda)$, the transmission spectrum of the used filter (Figure 3 a). In addition, the optical density for the full optical path length through the hemi sphere demonstrates transmission of T_{520nm} = 90% at a detection wavelength at 520 nm (Supplementary Figure 5). Again, this value already contains reflection losses at the glass/air transition. Finally, a detected photon at 520 nm has only $\Phi_{Photonenergy}$ = 375 nm/520nm = 72,11% of the energy of a 375 nm excitation photon. Thus, for a combined energy transfer and fluorescence quantum yield of 100% one would expect to detect on average a power of

$$I_{Exc} \cdot T_{375nm} \cdot \Phi_{Abs} \cdot \Phi_{Photonenergy} \cdot T_{520nm} \cdot T_{Detection} \cdot F_{Area} = \tag{4}$$

$$94\ \mu W \cdot 0.83 \cdot 0.997 \cdot 0.72 \cdot 0.9 \cdot 0.44 \cdot 0.00105 = 23.3\ nW.$$

The average three-dimensional weighed power is with 19.3 nW (Supplementary Figure 2 and Supplementary Table 2) very close to that value, indicating very high combined energy transfer and fluorescence quantum yields of 19.3 nW/23.3 nW = 83%. This observation is supporting a higher fluorescence quantum yield of Coumarin 6 than 80% as already indicated in Literature[7]. In addition, delayed singlet luminescence due to triplet-triplet annihilation[8] might additionally contribute to the observed intensities, as described in more detail in the main text.

Finally, the absorption spectrum in Supplementary Figure 6 also demonstrates that a single foil only re-absorbs 0.5% of in the emission maximum of the re-directed acceptor emission (~525 nm, Figure 3 a).

Supplementary Note 5

Energy transfer efficiency assessed from experimentally observed energy transfer rates.

In the experimental pump-probe experiments rise time constants in the acceptor spectral region of 20 ps and ~200 ps were observed upon donor excitation. No such rise was observed in identical measurements using acceptor only. The first value is on a similar order as the fast time constant estimated from the Förster calculations for the nearest donor-acceptor distance. The second times scales likely reflects energy transfer from more distant donors as well as intra-donor energy migration and dipole re-orientation (Figure 3 c). Both time constants are significantly smaller than the lifetime of the donor of $\tau_{S_1}^{D} = 3\ \mathrm{ns}$, indicating again a high energy transfer quantum efficiency.

Supplementary Note 6

Energy transfer efficiency determined from comparing absorption with fluorescence excitation spectra

Another common way to determine energy transfer efficiencies in donor acceptor systems in liquid solution is to compare the fraction of absorbed light, as determined from absorption spectra with fluorescence excitation spectra. However, a closer look in the case of donor and aligned acceptor dyes immobilized in a polymer foil shows that here a precise determination is more difficult when using standard fluorometers and absorption spectrometers. Different energy transfer de-polarization and photoselection effects in the donor and acceptor spectral excitation range as well as different refraction and total internal reflection escape cone losses significantly affect the finally detected intensity at different angles. For example, photoselection and air-foil refraction leads to excitation of acceptors that preferentially re-emit in certain directions while de-polarization after excitation of donors and subsequent energy transfer to the acceptors lead rather to emission for isotropically oriented acceptors. This is further complicated for different donor and acceptor orientation distribution, as in the case of our samples with aligned acceptors. The polarization sensitive excitation spectra of such a foil in Supplementary Figure 7 a is illustrating this. Here, almost the same acceptor emission intensity is observed when exciting in the donor absorption range with vertically or horizontally polarized light while drastically different intensities are observed for vertically or horizontally excitation in the acceptor absorption range. This makes it difficult to accurately compare the excitation with the absorption spectra for an estimate of the energy transfer quantum efficiencies in these samples. In addition, it is not clear to what extent standard absorption spectrometers and fluorescence spectrometer are insensitive to different excitation and detection polarization properties of the sample. It has also to be considered that the foils needs to be oriented with some angle, e.g. 45°, in the excitation and detection beams in the fluorescence spectrometer while they are oriented in 90° in the absorption spectrometer. Therefore, we believe that the direct power measurements described above give a more accurate read-out for the combined energy

transfer and acceptor fluorescence quantum efficiency. Nevertheless, when using no polarizing element in the excitation and detection path of the fluorescence spectrometer as well as absorption spectrometer a comparison of the spectra still indicates energy transfer quantum efficiencies of at least 80% for the foils with aligned acceptors (Supplementary Figure 7 b). Here, the procedure described in Gutierrez et al.[1] was used. Please note that in the case of highly concentrated samples not the measured absorbance (Supplementary Figure 6) can be used for that comparison but rather the absorptance, which is linearly related to the fraction of absorbed light (Supplementary Figure 7 b)[1] . The absorptance can be directly calculated from the absorbance[1]. We also note, that the calculated quantum efficiency upon excitation in spectral ranges 300 nm, which has increasing contributions from direct acceptor absorption, is decreasing instead of increasing. As direct excitation of acceptor should rather result in 100% efficiency values this observation further supports the assumption that efficiencies determined by direct power measurements are more accurate. A more precise polarization sensitive comparison of excitation spectra with absorption spectra will subject of future studies.

Supplementary Note 7

Summary and Re-absorption losses after multiple foil transits

In summary, a single foil harvests nearly 100% of the excitation light and the re-direction quantum efficiency itself is ~90% when assuming materials with refractive indices of 1.5 or 1.9 for total internal reflection wave guiding (Supplementary Note 2). Energy transfer quantum efficiencies in the range between 80% to 100% have been observed with various approaches (Supplementary Note 2, Supplementary Note 3, Supplementary Note 4, Supplementary Note 5, Supplementary Note 6). A single foil only re-absorbs 0.5% of in the emission maximum of the re-directed acceptor emission (Supplementary Note 4).Even after multiple transitions through foils and reflections still a major part of the re-directed photons would not be lost since re-absorption leads in at least 78% (Supplementary Table 1) of the cases again to re-emission in favorable directions. For example, even after 20 foil transits $(1-0.005\cdot 0.78)^{20} = 92\%$ of photons in the acceptor emission maximum are either transmitted or re-emitted and can still be directed towards a photovoltaic device.

Supplementary References

1. Gutierrez, G.D., Coropceanu, I., Bawendi, M.G. & Swager, T.M. A Low Reabsorbing Luminescent Solar Concentrator Employing Pi-Conjugated Polymers. *Adv. Mater.* **28,** 497–501 (2016).
2. Reynolds, G.A. & Drexhage, K.H. New coumarin dyes with rigidized structure for flashlamp-pumped dye lasers. *Optics Communications* **13,** 222–225 (1975).
3. Olmsted, J. Calorimetric determinations of absolute fluorescence quantum yields. *J. Phys. Chem.* **83,** 2581–2584 (1979).
4. Jones, G., Jackson, W.R., Choi, C.Y. & Bergmark, W.R. Solvent effects on emission yield and lifetime for coumarin laser dyes. Requirements for a rotatory decay mechanism. *J. Phys. Chem.* **89,** 294–300 (1985).
5. Specified by the manufacturer (www.atto-tec.com).
6. Kubin, R.F. & Fletcher, A.N. Fluorescence quantum yields of some rhodamine dyes. *Journal of Luminescence* **27,** 455–462 (1982).
7. Debije, M.G. & Verbunt, P.P.C. Thirty Years of Luminescent Solar Concentrator Research: Solar Energy for the Built Environment. *Adv. Energy Mater.* **2,** 12–35 (2012).
8. Nickel, B. & Karbach, H.-J. Complete spectra of the delayed luminescence from aromatic compounds in liquid solutions. On the observability of direct radiative triplet-triplet annihilation. *Chemical Physics* **148,** 155–182 (1990).
9. Lakowicz, J.R. *Principles of fluorescence spectroscopy.* 3rd ed. (Springer, New York, N.Y, op. 2006).
